# Sex-related differences in presentation, treatment, and outcomes of Asian patients with atrial fibrillation: a report from the prospective APHRS-AF Registry

**DOI:** 10.1038/s41598-023-45345-3

**Published:** 2023-10-26

**Authors:** Tommaso Bucci, Alena Shantsila, Giulio Francesco Romiti, Wee-Siong Teo, Hyung-Wook Park, Wataru Shimizu, Davide Antonio Mei, Hung-Fat Tse, Marco Proietti, Tze-Fan Chao, Gregory Y. H. Lip, Asia-Pacific Heart Rhythm Society Atrial Fibrillation Registry Investigators, Chun-Wah Siu David, Wataru Shimizu, Kenji Yodogawa, Hiroyuki Tsutsui, Yasushi Mukai, Hirofumi Tomita, Daisuke Horiuchi, Joji Hagii, Kazutaka Aonuma, Yasuo Okumura, Masahiko Goya, Kenzo Hirao, Masayoshi Ajioka, Nobuhisa Hagiwara, Atsushi Suzuki, Teiichi Yamane, Takanori Ikeda, Hitomi Yuzawa, Kazuhiro Satomi, Yoshinao Yazaki, Keiichi Fukuda, Yoshinori Kobayashi, Norishige Morita, Toyoaki Murohara, Eiichi Watanabe, Masahide Harada, Satoru Sakagami, Takahiro Saeki, Kengo Kusano, Koji Miyamoto, Shinsuke Miyazaki, Hiroshi Tada, Koichi Inoue, Nobuaki Tanaka, Yukihiro Koretsune, Haruhiko Abe, Yasuki Kihara, Yukiko Nakano, Akihiko Shimizu, Yasuhiro Yoshiga, Tomohiro Sakamoto, Ken Okumur, Naohiko Takahashi, Tetsuji Shinohara, Kyoko Soejima, Masahiko Takagi, Mitsuharu Kawamura, Yumi Munetsugu, Sung-Hwan Kim, Jae-Min Shim, Jae Sun Uhm, Sung Il Im, Hyoung-Seob Par, Jun Hyung Kim, Young Keun On, Il-Young Oh, Seung Yong Shin, Jum Suk Ko, Jun Beom Park, Wee-Siong Teo, Kelvin Cheok-Keng Won, Toon-Wei Lim, David Foo, Shih-Ann Chen, Shih-Ann Chen, Tze-Fan Chao, Yenn-Jiang Lin, Fa-Po Chung, Yu-Feng Hu, Shil-Lin Chang, Ta-Chuan Tuan, Jo-Nan Liao, Cheng-Hung Li, Jin-Long Huang, Yu-Cheng Hsieh, Tsu-Juey Wu, Ying-Chieh Liao, Cheng-Hung Chiang, Hsiang-Chiang Hsiao, Tung-Chen Yeh, Wei-Siang Lin, Wen-Yu Lin, Jen-Yuan Kuo, Chong-Lie Hong, Yih-Je Wu, Ying-Siang Li, Jui-Peng Tsai, Kuo-Tzu Sung, Sheng-Hsiung Chang

**Affiliations:** 1 Liverpool Centre of Cardiovascular Science at University of Liverpool, Liverpool John Moores University and Liverpool Heart & Chest Hospital, Liverpool, UK; 2 Department of General and Specialized Surgery, Sapienza University of Rome, Rome, Italy; 3 Department of Translational and Precision Medicine, Sapienza University of Rome, Rome, Italy; 4 Department of Cardiology, National Heart Centre, Singapore, Singapore; 5 Department of Cardiovascular Medicine, Chonnam National University Hospital, Gwangju, Korea; 6 Department of Cardiovascular Medicine, Nippon Medical School, Tokyo, Japan; 7 Cardiology Division, Department of Biomedical, Metabolic and Neural Sciences, University of Modena and Reggio Emilia, Policlinico Di Modena, Modena, Italy; 8 Division of Cardiology, Department of Medicine, School of Clinical Medicine, Queen Mary Hospital, the University of Hong Kong, Hong Kong SAR, China; 9 Department of Clinical Sciences and Community Health, University of Milan, Milan, Italy; 10 Division of Subacute Care, IRCCS Istituti Clinici Scientifici Maugeri, Milan, Italy; 11 Institute of Clinical Medicine, and Cardiovascular Research Center, National Yang Ming Chiao Tung University, Taipei, Taiwan; 12 Division of Cardiology, Department of Medicine, Taipei Veterans General Hospital, No. 201, Sec. 2, Shih-Pai Road, Taipei, Taiwan; 13 Danish Center for Health Services Research, Department of Clinical Medicine, Aalborg University, Aalborg, Denmark; 14 Liverpool Centre for Cardiovascular, Science William Henry Duncan Building 6 West Derby Street, Liverpool, L7 8TX UK; 15 Queen Mary Hospital, Hong Kong, China; 16 Department of Cardiovascular Medicine, Graduate School of Medicine, Nippon Medical School, Tokyo, Japan; 17 Department of Cardiovascular Medicine, Faculty of Medical Sciences, Kyushu University, Kyushu, Japan; 18 Department of Cardiology, Hirosaki University Graduate School of School of Medicine, Hirosaki, Japan; 19 Hirosaki Stroke and Rehabilitation Center, Hirosaki, Japan; 20 Division of Cardiology, University of Tsukuba Hospital, Tsukuba, Japan; 21 Division of Cardiology, Nihon University Itabashi Hospital, Itabashi, Japan; 22 Department of Cardiovascular Medicine, Tokyo Medical and Dental University, Tokyo, Japan; 23 Division of Cardiology, Tosei General Hospital, Seto, Japan; 24 Department of Cardiology, Tokyo Women’s Medical University, Tokyo, Japan; 25 Department of Cardiovascular Medicine, Jikei University, Tokyo, Japan; 26 Faculty of Medicine), Toho University, Tokyo, Japan; 27 Heart Rhythm Center, Tokyo Medical University, Tokyo, Japan; 28 Department of Cardiology, Keio University School of Medicine, Tokyo, Japan; 29 Division of Cardiology, Tokai University Hachioji-Hospital, Tokyo, Japan; 30 Department of Cardiology, Nagoya University, Tokyo, Japan; 31 Department of Cardiology, Fujita Health University School of Medicine, Tokyo, Japan; 32 National Hospital Organization Kanazawa Medical Center, Tokyo, Japan; 33 Department of Cardiovascular Medicine, National Cerebral and Cardiovascular Center, Osaka, Japan; 34 Department of Cardiovascular Medicine, Faculty of Medical Sciences, University of Fukui, Fukui, Japan; 35 Cardiovascular Center, Sakurabashi Watanabe Hospital, Osaka, Japan; 36 National Hospital Organization Osaka National Hospital, Osaka, Japan; 37 Department of Cardiovascular Medicine, Hiroshima University Graduate School of Biomedical and Health Sciences, Hiroshima, Japan; 38 Department of Medicine and Clinical Science, University Graduate School of Medicine, Tokyo, Japan; 39 Division of Cardiology, Saiseikai Kumamoto Hospital Cardiovascular Center, Kumamoto, Japan; 40 Oita University Hospital, Yufu, Japan; 41 Department of Cardiovascular Medicine, Kyorin University School of Medicine, Tokyo, Japan; 42 Kansai Medical University Medical Center, Tokyo, Japan; 43 Division of Cardiology, Showa University School of Medicine, Tokyo, Japan; 44 Division of Cardiology, Department of Internal Medicine, College of Medicine, Seoul St. Mary’s Hospital, The Catholic University of Korea, Seoul, South Korea; 45 Division of Cardiology, Korea University College of Medicine and Korea University Medical Center, Seoul, South Korea; 46 Division of Cardiology, Department of Internal Medicine, Yongin Severance Hospital, Yonsei University College of Medicine, Yongin, Korea; 47 Division of Cardiology, Department of Internal Medicine, Kosin University Gospel Hospital, Kosin University College of Medicine, Busan, Korea; 48 Division of Cardiology, Department of Internal Medicine, Keimyung University Dongsan Hospital, Daegu, South Korea; 49 Department of Cardiology, Chungnam National University, Daejeon, South Korea; 50 Division of Cardiology, Department of Medicine, Heart Vascular and Stroke Institute, Samsung Medical Center, Sungkyunkwan University School of Medicine, Seoul, South Korea; 51 Division of Cardiology, Department of Internal Medicine, Seoul National University Bundang Hospital, Seoul, South Korea; 52 Cardiovascular & Arrhythmia Centre, Chung-Ang University Hospital, Chung-Ang University, Seoul, Korea; 53 Division of Cardiology, Department of Internal Medicine, Wonkwang University School of Medicine, Iksan, Korea; 54 Department of Cardiology, College of Medicine, Ewha Womans University, Seoul, Korea; 55 National Heart Centre Singapore, Singapore, Singapore; 56 Changi General Hospital, Singapore, Singapore; 57 National University Hospital, Singapore, Singapore; 58 Tan Tock Seng Hospital, Singapore, Singapore; 59 Taichung Veterans General Hospital, Taichung, Taiwan; 60 Taipei Veterans General Hospital, Taipei, Taiwan; 61 Kaohsiung Veterans General Hospital, Kaohsiung, Taiwan; 62 Tri-Service General Hospital, Taipei, Taiwan; 63 Mackay Memorial Hospital, Taipei, Taiwan

**Keywords:** Cardiology, Medical research

## Abstract

We aimed to investigate the sex-related differences in the clinical course of patients with Atrial Fibrillation (AF) enrolled in the Asia–Pacific-Heart-Rhythm-Society Registry. Logistic regression was utilized to investigate the relationship between sex and oral anticoagulant, rhythm control strategies and the 1-year chance to maintain sinus rhythm. Cox-regression was utilized to assess the 1-year risk of all-cause, and cardiovascular death, thromboembolic events, acute coronary syndrome, heart failure, and major bleeding. In the whole cohort (4121 patients, 69 ± 12 years,34.3% female), females had different cardiovascular risk factors, clinical manifestations, and disease perceptions than men, with more advanced age (72 ± 11 vs 67 ± 12 years, p < 0.001) and dyslipidemia (36.7% vs 41.7%, p = 0.002). Coronary artery disease was more prevalent in males (21.1% vs 16.1%, p < 0.001) as well as the use of antiplatelet drugs. Females had a higher use of oral anticoagulant (84.9% vs 81.3%, p = 0.004) but this difference was non-significant after adjustment for confounders. On multivariable analyses, females were less often treated with rhythm control strategies (Odds Ratio [OR] 0.44,95% Confidence Interval [CI] 0.38–0.51) and were less likely to maintain sinus rhythm (OR 0.27, 95% CI 0.22–0.34) compared to males. Cox-regressions analysis showed no sex-related differences for the risk of death, cardiovascular, and bleeding. The clinical management of Asian AF patients should consider several sex-related differences.

## Introduction

Atrial fibrillation (AF) is one of the most common arrhythmias worldwide and is associated with an increased risk of cardiovascular events and death^1^. In patients with AF several differences in terms of clinical presentation, therapeutical management, and long-term outcomes are related to sex^2,3^. Females are often more symptomatic, have a higher prevalence of comorbidities and a higher risk of thromboembolic events compared to males^2–4^. The mechanisms responsible for these differences are unknown, yet previous studies suggest the involvement of anthropometric, hormonal, structural, and electrophysiological factors^5^. However, most studies that investigated sex differences in patients with AF have been conducted in Western populations whereas less information is available about their generalizability to other ethnic groups. Indeed, the features of cardiovascular diseases not only differ between sexes but can also differ between ethnic groups within the same sex^6^.

To date, only a few studies investigated these aspects in Asia–Pacific populations^7–10^, and there is still a need for prospective data on sex-related differences in presentation, treatment, and outcomes of Asian patients with AF. In 2015 the Asia–Pacific Heart Rhythm Society (APHRS) in collaboration with the European Society of Cardiology (ESC) started a registry in five different Asian countries (Hong Kong, Singapore, South Korea, Japan, and Taiwan) to collect prospective contemporary data regarding the management and the clinical outcomes of AF patients.

The aim of the present study is to investigate the presence of sex-related differences in terms of clinical presentation, medical treatment, and long-term outcomes in a large prospective cohort of Asian patients with AF enrolled in the APHRS registry.

## Methods

The study protocol for patients’ enrolment and data collection was the same of the ESC—European Heart Rhythm Association (EHRA) EURObservational Research Programme in AF General Long-Term (EORP-AF) Registry, as reported previously^11^. The population comprised consecutive in- and outpatients with AF who had undergone a cardiology examination in tertiary and general hospitals in five Asian-Pacific countries (Hong Kong, South Korea, Japan, Singapore, and Taiwan). Enrolment into the registry started in 2015, and the end of enrolment was in 2017. All eligible patients had an electrocardiogram (ECG) documenting AF within 1 year before the first visit and signed a written informed consent according to the declaration of Helsinki and the local regulations. After the baseline clinical assessment, a 1-year follow-up was performed by the local investigators. The study protocol was approved by the local ethics committee and was registered on ClinicalTrials.gov (NCT04807049).

### Clinical scores

The CHA_2_DS_2_-VASc score was calculated as follows: congestive heart failure (1 point); hypertension (1 point); age 65–74 (1 point) and > 75 years (2 points); diabetes (1 point); stroke (2 points); vascular disease (1 point); and female sex category (1 point)^12^.

HAS-BLED score was calculated as follows: uncontrolled hypertension (1 point), abnormal renal or liver function (defined as dialysis, renal transplant, serum creatinine > 200 mmol/L for the former and liver cirrhosis, bilirubin > 2 × upper limit of normal, aspartate aminotransferase/ alanine transaminase/ alkaline phosphatase > 3 × upper limit of normal for the latter, 1 point each); history of stroke (1 point); history of bleeding (1 point); labile international normalized ratio (INR) (1 point); age > 65 years (1 point); and drugs (e.g., aspirin or non-steroidal anti-inflammatory drugs or alcohol) (1 point)^13^.

Classification of AF-related symptoms was performed according to the EHRA score^14^ as follows: EHRA I, no symptoms; EHRA II, mild symptoms (normal daily activity not affected); EHRA III, severe symptoms (normal daily activity affected); EHRA IV, disabling symptoms (normal daily activity discontinued).

EHRA score considers symptoms attributable to AF and reverse or reduce upon restoration of sinus rhythm or with effective rate control and it was determined by recruiting sites.

EuroQoL is a well validated questionnaire utilized to evaluate the quality of life, that consists of five dimensions (mobility, self-care, usual activities, pain/discomfort and anxiety/depression) with five possible levels for each dimension (no problems, slight problems, moderate problems, severe problems and extreme problems). As previously reported, the answers provided by patients at baseline were utilized to generate a single numeric value for each domain that inversely related with the quality of life (highest value correspond to the worst quality of life)^15^.

### Rhythm control definitions

After the enrolment, all patients who received a rhythm control intervention such as electrical or pharmacological cardioversion, catheter ablation, or were prescribed an antiarrhythmic drug (Class Ia, Class Ic, Class III), were included in the ‘rhythm control’ group. All the other patients were considered as treated with rate control strategies.

### Statistical analysis

The distribution of linear variables was assessed by the Kolmogorov–Smirnov test. Continuous variables with normal distribution were expressed as mean ± standard deviation (SD) and compared by Student’s T test. Categorical variables were reported as counts and percentages and were compared with the χ^2^ test.

Logistic regression analysis was used to calculate Odds Ratios (OR) with relative 95% Confidence Interval (95% CI) for (i) oral anticoagulant (OAC) prescription, (ii) Vitamin K antagonist (VKA) use, (iii) rhythm control interventions (pharmacological and electrical cardioversion, and catheter ablation), and (iv) 1-year maintenance of sinus rhythm in patients with rhythm control strategies.

The incidence rate of adverse events (All-cause death, cardiovascular death, thromboembolic events, acute coronary syndrome or significant coronary artery disease requiring percutaneous coronary intervention (ACS/PCI), new or worsening of a preexisting heart failure, and major bleeding) was calculated as the number of events / total person-years ratio and reported as incidence for 100 persons/year with relative 95% CI. The 1-year risks of adverse events were compared between males and females. Cox proportional hazards regression time to the first event analysis was used to calculate the unadjusted and adjusted relative hazard ratios (HRs) and 95% CI of adverse events. All the multivariable Cox regression analyses were adjusted for the following covariates: age, CHA_2_DS_2_-VASc or HAS-BLED risk scores, OAC, chronic kidney disease (CKD), paroxysmal AF, cancer, dementia, dyslipidemia, and chronic obstructive pulmonary disease (COPD). Proportional hazard assumptions were checked with the Schoenfeld residuals test. Patients without available data to calculate the clinical scores, or to investigate the antithrombotic treatment, the rhythm or rate management, or without follow-up were excluded from the analysis. All tests were 2-tailed, and analyses were performed using computer software packages (SPSS-25.0, SPSS Inc., Chicago, IL). A p-value < 0.05 was considered as statistically significant.

## Results

Of the 4666 patients with AF enrolled in the APHRS registry, 2 patients died before discharge, 458 were lost to follow-up or withdrawn their informed consent and 85 had an unknown follow-up status. Thus, in the final analysis, we considered 4121 patients with available follow-up, of whom 1423 (34.5%) were females (mean age 71.5 ± 11.2 years) and 2698 (65.5%) were males (mean age 67.0 ± 11.1 years).

### Clinical characteristics

Females were older, with a higher prevalence of dyslipidemia, dementia and anemia, a lower prevalence of coronary artery disease (CAD) and COPD, and were more frequently treated with statins, digoxin, diuretics, and calcium antagonist than males (Table 1). The most frequent AF patterns were paroxysmal in females and persistent in males. Female sex was associated with a higher prevalence of severe or disabling symptoms (EHRA score III or IV), mainly represented by palpitations and chest pain, and worse quality of life, as shown by the higher EuroQoL scores in all five domains (Table 1).

**Table 1:** Baseline characteristics of patients according to sex.

	Males n = 2698	Females n = 1423	p-value
Age (years)	67.0 ± 11.90	71.5 ± 11.17	< 0.001
Age ≥ 75 years	754 (28.0)	605 (42.5)	< 0.001
Systolic blood pressure (mmHg)	128 ± 18	130 ± 19	0.002
Diastolic blood pressure (mmHg)	75 ± 12	73.0 ± 12	< 0.001
Heart rate (bpm)	77 ± 16	77 ± 17	0.533
BMI (Kg/m^2^)	25.1 ± 3.9	24.9 ± 4.8	0.179
AF pattern (n = 4108)			
First Diagnosed, n (%)	180 (6.7)	112 (7.9)	
Paroxysmal, n (%)	1094 (40.7)	628 (44.2)	
Persistent, n (%)	697 (25.9)	284 (20.0)	< 0.001
Long-standing persistent, n (%)	276 (10.3)	138 (9.7)	
Permanent, n (%)	441 (16.4)	258 (18.2)	
Concomitant disease			
Hypertension, n (%)	1616 (60.3)	892 (63.1)	0.077
CAD, n (%)	561 (21.1)	225 (16.1)	< 0.001
HF, n (%)	547 (20.5)	311 (22.2)	0.205
NYHA I, n (%)	231 (42.2)	119 (38.3)	
NYHA II, n (%)	247 (45.2)	131 (42.1)	0.023
NYHA III, n (%)	63 (11.5)	51 (16.4)	
NYHA IV, n (%)	6 (1.1)	10 (3.2)	
Diabetes, n (%)	635 (23.8)	364 (26.1)	0.114
Lipid disorder, n (%)	974 (36.7)	585 (41.7)	0.002
Smoker, n (%)	321 (11.9)	30 (2.1)	0.001
Previous Stroke/TIA, n (%)	247 (9.2)	148 (10.5)	0.194
Previous bleedings, n (%)	195 (7.3)	116 (8.2)	0.277
ICH, n (%)	44 (1.6)	26 (1.8)	0.637
Major extracranial bleeding, n (%)	89 (3.3)	41 (2.9)	0.473
PAD, n (%)	39 (1.5)	13 (0.9)	0.143
CKD, n (%)	203 (7.5)	109 (7.7)	0.876
Liver disease	125 (4.7)	59 (4.2)	0.468
COPD, n (%)	99 (3.7)	14 (1.0)	< 0.001
Cancer, n (%)	61 (2.3)	34 (2.4)	0.794
Dementia, n (%)	31 (1.2)	42 (3.0)	< 0.001
Anemia, n (%)	169 (6.3)	126 (8.9)	0.002
Medications			
ACE-I	381 (14.2)	157 (11.1)	0.005
ARBs	696 (25.9)	377 (26.6)	0.617
Beta Blockers	1346 (50.1)	731 (51.7)	0.346
Statins	964 (35.9)	582 (41.2)	0.001
Oral antidiabetics	428 (15.9)	229 (16.2)	0.810
Insulin	63 (2.3)	39 (2.8)	0.418
Digoxin	246 (9.1)	215 (15.1)	< 0.001
Diuretics	575 (21.3)	330 (23.2)	0.041
Aldosterone blockers	176 (6.5)	93 (6.5)	0.161
Calcium channel blockers	605 (22.4)	352 (24.7)	0.021
Calcium channel blockers non-DHP	254 (9.4)	316 (22.2)	< 0.001
PPIs	821 (30.4)	366 (25.7)	0.001
Symptomatic status			
EHRA I, n (%)	1780 (66.0)	866 (60.9)	
EHRA II, n (%)	771 (28.6)	443 (31.1)	0.001
EHRA III, n (%)	130 (4.8)	101 (7.1)	
EHRA IV, n (%)	17 (0.6)	13 (0.9)	
Type of symptoms			
Palpitations, n (%)	578 (21.4)	353 (24.8)	0.014
Syncope, n (%)	37 (1.4)	26 (1.8)	0.257
Shortness of breath, n (%)	271 (10.0)	171 (12.0)	0.052
Chest pain, n (%)	141 (5.2)	102 (7.2)	0.012
General non-wellbeing, n (%)	56 (2.1)	17 (1.2)	0.042
Dizziness, n (%)	179 (6.6)	116 (8.2)	0.072
Fatigue, n (%)	110 (4.1)	55 (3.9)	0.741
Fear/Anxiety, n (%)	44 (1.6)	27 (1.9)	0.532
Other, n (%)	39 (1.4)	22 (1.5)	0.799
EuroQoL			
Mobility	1.24 ± 0.60	1.51 ± 0.88	< 0.001
Self-care	1.10 ± 0.45	1.27 ± 0.76	< 0.001
Usual activities	1.18 ± 0.53	1.41 ± 0.84	< 0.001
Pain/discomfort	1.34 ± 0.60	1.56 ± 0.77	< 0.001
Anxiety/depression	1.30 ± 0.62	1.47 ± 0.73	< 0.001
Thrombotic and hemorrhagic risk			
CHA_2_DS_2_-VASc	2.2 ± 1.6	3.6 ± 1.6	< 0.001
CHA_2_DS_2_-VASc ≥ 2	1730 (64.1)	1283 (90.2)	< 0.001
HAS-BLED	1.3 ± 1.0	1.5 ± 1.0	< 0.001
HAS-BLED ≥ 3	346 (12.8)	221 (15.5)	0.016
Antithrombotic treatment			
Oral anticoagulation, n (%)	2194 (81.3)	1208 (84.9)	0.004
VKA, n (%)	526 (24.0)	300 (24.8)	0.576
NOAC, n (%)	1668 (76.0)	908 (75.2)	
Dabigatran, n (%)	319 (11.8)	172 (12.1)	0.804
Rivaroxaban, n (%)	609 (22.6)	293 (20.6)	0.143
Apixaban, n (%)	468 (17.3)	293 (20.6)	0.011
Edoxaban, n (%)	272 (10.1)	150 (10.5)	0.644
Antiplatelet, n (%)	459 (17.0)	157 (11.0)	< 0.001
Aspirin mono, n (%)	320 (69.7)	109 (69.4)	
Other mono, n (%)	105 (22.9)	36 (22.9)	0.995
Dual, n (%)	34 (7.4)	12 (7.6)	
OAC + Antiplatelet, n (%)	459 (17.0)	157 (11.0)	< 0.001
Reasons for not using any OAC (n = 720)	n = 507	n = 215	
No indication (low risk), n (%)	237 (47.0)	81 (37.7)	0.021
Unwilling to take any OAC, n (%)	84 (16.7)	32 (14.9)	0.552
Prior bleeding, n (%)	17 (3.4)	14 (6.5)	0.058
OAC not considered adequate by physician despite stroke risk, n (%)	7 (1.4)	3 (1.4)	0.995
Recent / planned surgery / intervention, n (%)	16 (3.2)	12 (5.6)	0.127
Active peptic ulcer, n (%)	2 (0.4)	3 (1.4)	0.140
Anemia, n (%)	18 (3.6)	18 (8.4)	0.007
Thrombocytopenia, n (%)	2 (0.4)	2 (0.9)	0.379
Renal dysfunction, n (%)	14 (2.8)	12 (5.6)	0.065
Liver disease, n (%)	3 (0.6)	0 (0.0)	0.257
Malignancy, n (%)	8 (1.6)	2 (0.9)	0.491
Alcohol or drug abuse or psychosocial issues, n (%)	2 (0.4)	0 (0.0)	0.355
Frequent falls, n (%)	3 (0.6)	8 (3.7)	0.002
Dementia, n (%)	0 (0.0)	2 (0.9)	0.030
Recent stroke, n (%)	2 (0.4)	2 (0.9)	0.379
Intolerance / allergy, n (%)	2 (0.4)	1 (0.5)	0.897
Other, n (%)	88 (17.4)	23 (10.7)	0.001

*BMI* body mass index, *AF* atrial fibrillation, *CAD* coronary artery disease, *HF* heart failure, *NYHA* New York Heart Association (classification), *TIA* transient ischemic attack, *ICH* intracranial hemorrhage, *PAD* peripheral artery disease, *CKD* chronic kidney disease, *COPD* chronic obstructive pulmonary disease, *ACE-I* angiotensin converting enzyme inhibitors, *ARBs* angiotensin receptor blockers, *DH*: dihydropyridine, *PPIs* proton pump inhibitors, *EHRA* European Heart Rhythm Association, *VKA* vitamin-K antagonist, *NOAC* non vitamin-K antagonist anticoagulant, *OAC* oral anti-coagulant.

### Antithrombotic management

Females had higher mean CHA_2_DS_2_-VASc and HAS-BLED risk scores, greater use of OAC, and were less treated with antiplatelets drugs compared to males (Table 1). In anticoagulated patients, no significant sex-related differences were found for the relative prevalence of VKA and non-vitamin K antagonist oral anticoagulants (NOAC) use. The main reasons associated with the lack of any OAC therapy were due to anemia, frequent falls, and dementia in females, and a low thromboembolic risk score (CHA_2_DS_2_-VASc < 1) in males (Table 1).

**Figure 1:**
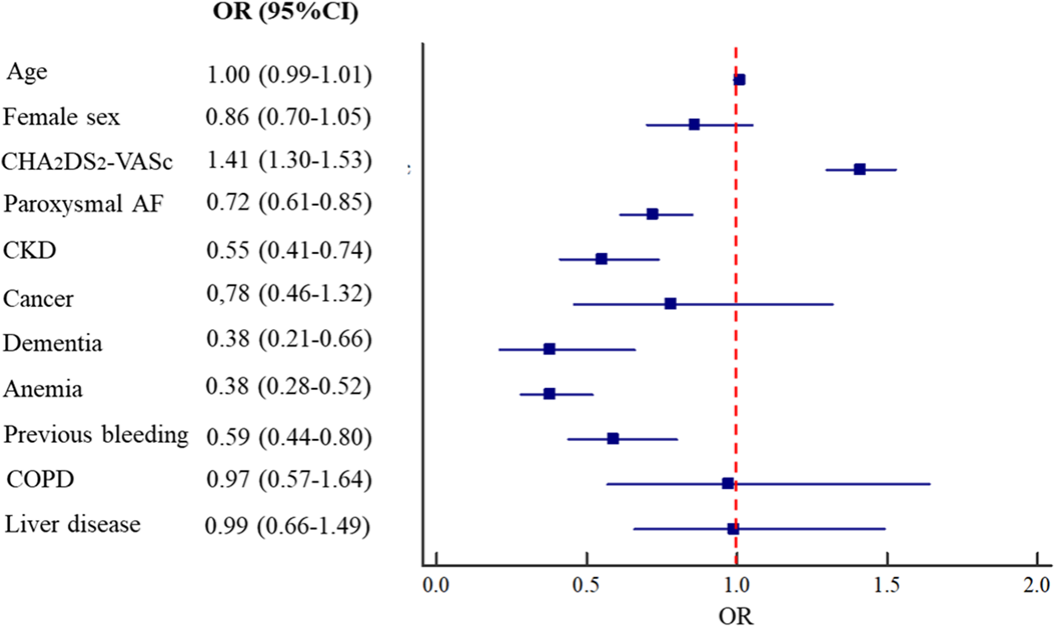
Logistic multivariate analysis for factors associated with oral anticoagulant use.

Given the baseline higher prevalence of OAC use in females, we investigated this aspect using multivariate regression analysis (Fig. 1). The only factor independently associated with OAC use was higher CHA_2_DS_2_-VASc score, while paroxysmal AF, CKD, dementia, anemia and previous bleeding were associated with a lower OAC prescription. No significant associations between sex and OAC was found after adjustment for confounding factors (Fig. 1).

In our cohort, the most used OAC treatment was represented by NOAC and only 826 (24.3%) patients were treated with VKA. To investigate the presence of sex-related differences in OAC type, a multivariable logistic regression analysis showed age, CHA_2_DS_2_-VASc, CKD, and anemia were associated with a higher VKA use while paroxysmal AF and active cancer were associated with a lower VKA use. The lack of independent association between sex and VKA was confirmed also in this analysis (Supplementary Table 1).

### Rhythm control strategies and 1-year sinus rhythm maintenance

**Figure 2:**
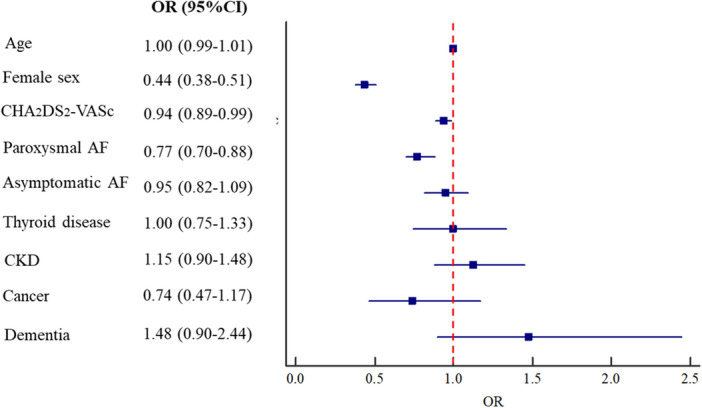
Logistic multivariate analysis for the factors associated with rhythm control strategies after the enrollment.

After the enrollment, 1561 (37.9%) patients were treated according to rhythm control strategies and 2560 (62.1%) with rate control approaches. Overall, rhythm control strategies were less used in females than males (24.9% vs 45.1%, p < 0.001. Table 2), confirmed also in a logistic multivariate regression analysis adjusting for age, CHA_2_DS_2_-VASc, paroxysmal AF, asymptomatic AF, thyroid disease, CKD, cancer, and dementia (OR 0.44, 95% CI 0.38–0.51, Fig. 2).

**Table 2:** Rhythm control strategies in patients with atrial fibrillation according to sex.

	Males n = 1208	Females n = 353	p-value
Antiarrhythmics, n (%)	672 (55.6)	265 (75.1)	< 0.001
Amiodarone, n (%)	266 (39.6)	61 (23.0)	< 0.001
Dronedarone, n (%)	68 (10.1)	31 (11.7)	0.479
Flecainide, n (%)	142 (21.1)	42 (15.8)	0.067
Propafenone, n (%)	195 (29.0)	100 (37.7)	0.010
Sotalol, n (%)	39 (5.8)	33 (12.5)	0.001
Disopyramide, n (%)	4 (0.6)	2 (0.8)	0.783
Quinidine, n (%)	1 (0.0)	0 (0.0)	0.530
Interventional procedures, n (%)	770 (63.7)	104 (29.5)	< 0.001
Electrical cardioversion, n (%)	151 (12.5)	26 (7.4)	0.007
Pharmacological cardioversion, n (%)	145 (12.0)	68 (19.3)	< 0.001
Catheter ablation, n (%)	594 (49.2)	19 (5.4)	< 0.001

In the rhythm control group, the procedures most used in males were electrical cardioversion and catheter ablation while females were more commonly managed with antiarrhythmic therapies and pharmacological cardioversion (Table 2).

**Figure 3:**
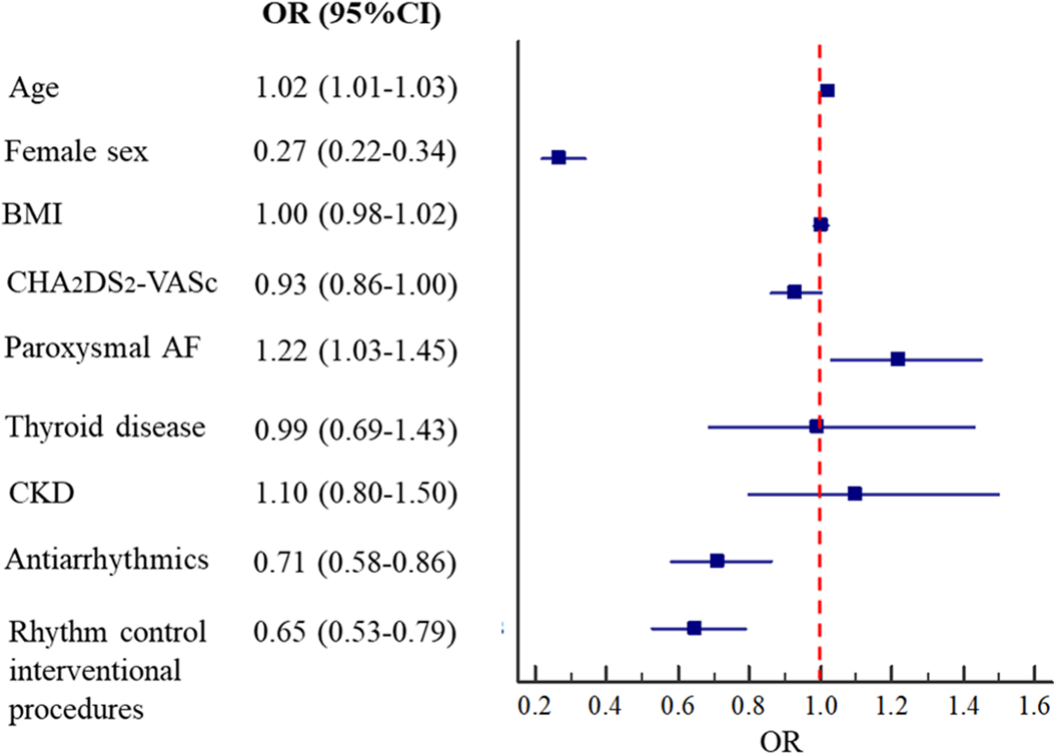
Logistic multivariate analysis for factors associated with the sinus rhythm maintenance after 1-year of follow-up.

After 1-year of follow-up of the 1561 patients in the rhythm control group, only 1082 (69.3%) had an electrocardiogram attesting to their rhythm as follows: 440 (40.7%) were in sinus rhythm, 566 (52.3%) in AF, 8 (0.7%) in atrial flutter, 38 (3.5%) had a pacemaker rhythm and 30 showed other types of arrhythmias (2.8%). On multivariate analysis adjusted for age, Body Mass Index, CHA_2_DS_2_-VASc, paroxysmal AF, thyroid disease, CKD, antiarrhythmics, and rhythm control interventional procedures, female sex was independently associated with a lower chance to maintain the sinus rhythm (OR 0.27, 95% CI 0.22–0.34) compared to males (Fig. 3).

### Risk of adverse events during the 1-year follow-up.

In the whole cohort, after 1-year of follow-up the following events were reported: 118 (2.9%) all-cause death, 34 (0.8%) cardiovascular death, 28 (0.7%) thromboembolic events, 41 ACS/PCI (1.0%), 96 (2.3%) new/worsening heart failure, and 46 (1.1%) major bleedings. Females had a significantly higher incidence rate of cardiovascular death (p = 0.004) and thromboembolic events (p = 0.039) compared to males (Table 3).

**Table 3:** Incidence rates and Cox regression analyses for risk of primary and secondary outcomes according to sex.

	Number of events	Incidence rate 100 patients/year (95%CI)	p-value	Univariate HR (95% CI)	Multivariate* HR (95% CI)
All-cause death					
Male	68	2.6 (2.0–3.2)		Ref	Ref
Female	50	3.6 (2.7–4.7)	0.072	1.40 (0.97–2.02)	0.67 (0.44–1.01)
CV death					
Male	14	0.5 (0.3–0.9)		Ref	Ref
Female	20	1.4 (0.9–2.2)	0.004	1.63 (0.76–3.54)	0.80 (0.34–1.85)
Thromboembolic events					
Male	13	0.5 (0.3–0.8)		Ref	Ref
Female	15	1.1 (0.6–1.8)	0.039	2.19 (1.04–4.60)	1.75 (0.76–4.04)
ACS/PCI					
Male	29	1.1 (0.7–1.6)		Ref	Ref
Female	12	0.9 (0.4–1.5)	0.499	0.78 (0.40–1.53)	0.52 (0.25–1.08)
New/worsening HF					
Male	55	2.1 (1.6–2.7)		Ref	Ref
Female	41	3.0 (2.1–4.1)	0.088	1.40 (0.93–2.10)	0.95 (0.61–1.49)
Major bleeding					
Male	28	1.1 (0.7–1.5)		Ref	Ref
Female	18	1.3 (0.8–2.1)	0.494	1.23 (0.68–2.22)	1.05 (0.55–1.87)

*CI* confidence interval, *HR* hazard ratio, *CV* cardiovascular, *ACS/PCI* acute coronary syndrome/percutaneous coronary intervention, *HF* heart failure, *AF* atrial fibrillation, *OAC* oral anti-coagulant, *CKD* chronic kidney disease, *COPD* chronic obstructive pulmonary disease, *Ref* reference group.

*Adjusted for: age, paroxysmal AF, CHA_2_DS_2_-VASc or HAS-BLED (for major bleeding), OAC, CKD, cancer, dementia, dyslipidemia, COPD.

On univariate Cox-regression, only thromboembolic events were associated with the female sex (HR 2.19, 95% CI 1.04–4.60) while on multivariate analysis adjusted for age, CHA_2_DS_2_-VASc or HAS-BLED (for major bleeding), OAC, CKD, paroxysmal AF, cancer, dementia, dyslipidemia, and COPD, no significative association was found (Table 3, Supplementary Tables 2, 3, 4, 5, 6, 7). Nevertheless, a non-significative trend for a lower risk of all-cause mortality was found in females (HR: 0.67, 95% CI 0.44–1.01) compared to males. (Table 3).

## Discussion

In this large prospective cohort of Asian patients with AF, our principal findings were as follows: (i) females had a different cardiovascular risk factor profile, more disabling symptoms, and worse quality of life; (ii) females were less treated with rhythm control strategies and had a lower maintenance of sinus rhythm; and (iii) females were not associated with a higher risk of thromboembolic events after adjustment for confounding factors.

The cardiovascular risk profile of females was characterized by advanced age and a high prevalence of dyslipidemia, whereas for males, there was more prevalent CAD and COPD. These findings are in contrast to the distinctive sex-related characteristics reported in the EORP-AF registry, in which CAD and COPD were more frequent in females while hypertension was in males^2^. Although the observational nature of these registries could itself warrant these differences, another possible explanation could be provided by the higher overall prevalence of dyslipidemia and the less effective cholesterol control achieved with statins reported in Asians compared to Western populations^16–18^. Hence, female sex, rather than a typical cardiovascular risk factor, represents a risk modifier that increases the risk associated with other comorbidities, especially in older subjects where the protective role of female hormones is lacking^19^. Thus, the high prevalence of dyslipidemia in older Asian women with AF, rather than an occasional finding, could result from the interactions between advanced age, hormonal changes, and ethnic factors.

In our population, after adjustment for confounding factors, no significative sex-related differences were found for OAC or VKA use. This may reflect recent studies showing that NOAC introduction was associated with less bleeding in Asian women, resulting in the sex differences seen^20^. However, when analyzing the reasons behind the choice of not using any OAC, this was evident in males with low thrombotic risk, and in females by the presence of a frail phenotype characterized by anemia, frequent falls, and dementia. Having achieved the large OAC use in most of the high-risk patients with AF, the next challenging step will be to find the best sex-based approaches to optimize OAC adherence and to avoid interruptions or discontinuation.

Based on previous studies, AF-related symptoms have a higher disabling effect in women than in males, leading to their worse quality of life^2,3,10,21^. Nevertheless, the possible explanation for this worst symptomatic status may simply lie in women’s older age, as well as rhythm control approaches utilized in our cohort.

The most recent guidelines for AF management, proposed by ESC^22^ and then also adopted by APHRS^23^, introduced the concept of the integrated ABC (Atrial fibrillation Better Care) pathway by which the management of the symptoms should be done according to the patient-centered symptom-directed decisions. Despite the impact of rhythm control strategy on mortality has been debated, but early rhythm control ameliorates AF-related symptoms and improves quality of life in patients who maintain sinus rhythm^24,25^. In the present study, we found that women, notwithstanding being more symptomatic, were less frequently treated with rhythm control approaches. In particular, females were associated with a lower use of electrical cardioversion, and catheter ablation procedures, and a higher use of antiarrhythmics drugs and pharmacological cardioversion compared to males. This is in line with other studies performed both in Asian^26,27^ and Western patients^3,21^. Of note, the different use of rhythm control strategies could also be related to the high rate of intra- and post-procedural complications, with the worse outcomes described in women^28–30^ as well as the high prevalence of CAD that could have contraindicated the use of antiarrhythmic drugs in males.

The observational nature of this study does not allow us to further clarify these issues, but better compliance with international guidelines for AF symptom management, could help not only to equalize the access to rhythm control procedures between sexes but especially to investigate, in future studies, if the mechanisms behind the low chance to maintain the sinus rhythm in women is associated with less effective approaches or intrinsic factors.

Analyzing the clinical outcome after 1-year of follow-up, we found that the female sex was associated with a higher incidence rate of cardiovascular death and thromboembolic events compared to males, yet this difference was non-significative after adjustment for confounding factors. Several studies performed in Western populations showed that female sex is a strong risk factor for stroke and thromboembolism^4,31,32^. However, in recent years growing evidence suggests that this relationship may be less evident when considering Asian populations^8,9,33^. In a Japanese population of 7406 patients with AF (29.2% females), after 2-years follow-up, no significative difference was found for the risk of stroke or thromboembolism (OR 1.24, 95% CI 0.83–1.86) in females compared to males^8^. Nonetheless there was a mix of OAC and non-OAC users, which does not account for quality of anticoagulation control if on a VKA, or label-adherent dosing in case of NOACs. This finding was further confirmed in a Taiwanese cohort of 7920 patients (45.8% females) followed for 4.5 years^33^, in a Korean cohort of 10,846 patients (46.8% women) followed for 2.8 years^9^, and in a Chinese cohort of 6239 patients (41.3% females) followed for 2.8 years^34^. One large metanalysis of more than 990,000 patients, demonstrated that the risk of stroke in women changes accordingly with the different ethnic group and was the lowest in Asians^35^.

The mechanisms responsible for sex differences in determining the risk of stroke in different ethnic groups are unclear. What is emerging is that sex should be considered as a dynamic risk modification factor that changes its relationship with the risk of cardiovascular diseases over time and based on the coexistence of other cardiovascular risk factors^19^. If in young people female sex has a protective role, in older age, it enhances the effect of other cardiovascular risk factors. Furthermore, the female sex could interact not only with the traditional cardiovascular risk factors but also with the novel characters involved in the global cardiovascular burden, as represented by the social determinants of health and ethnic origin.

### Limitations

Some limitations should be acknowledged when interpreting these results. First, this is a post-hoc analysis from an observational study, and caution should be used when generalizing our findings for due to the possible reduced power and presence of selection bias. Although we considered more than 88% of the initial cohort, the differences between the excluded and the included cohort, as well as the different prevalence of the two sexes in the final cohort, may have influenced the main analysis. The lack of exhaustive information regarding the type of catheter ablation intervention does not allow us to investigate the prevalence and the outcome associated with different types of procedures. Only 69.3% of the initial cohort have had an ECG attesting the rhythm after 1-year of follow-up and cannot be excluded that some patients may have had experienced a clinical silent AF paroxysm during the follow-up. Furthermore, no information is available about the time in therapeutic range in patients treated with VKA or about the dosage in those treated with NOAC, making it impossible to consider these factors in the survival analysis. Moreover, we had limited data on the impact of social determinants in this cohort, and further studies are needed to understand how gender-related factors and sex interact in determining the clinical phenotypes or the long-term outcomes of AF patients. Finally, the relatively small sample size, the short follow-up, and the small number of events could have affected the statistical power of our analysis missing to detect significant differences.

## Conclusion

Several sex-related differences should be considered as part of the management of Asian AF patients. Females were less likely to be treated with rhythm control strategies and were associated with a higher risk of AF recurrence.

### Supplementary Information

Supplementary Information.

## Data Availability

Data will be made available on request to the corresponding authors.
