# Supplementary material for: Sex-related differences in presentation, treatment, and outcomes of Asian patients with atrial fibrillation: a report from the prospective APHRS-AF Registry
Source: Sci Rep. 2023 Oct 26;13:18375. doi: 10.1038/s41598-023-45345-3 (PMC10603128; doi:10.1038/s41598-023-45345-3)
Supplement: Supplementary file 1 — Supplementary Information. [file 41598_2023_45345_MOESM1_ESM.docx]

**Sex-related differences in presentation, treatment, and outcomes of Asian patients with atrial fibrillation: A report from the prospective APHRS-AF Registry.**

Tommaso Bucci, Alena Shantsila, Giulio Francesco Romiti, Wee-Siong Teo, Hyung-Wook Park, Wataru Shimizu, Davide Antonio Mei, Hung-Fat Tse, Marco Proietti, Tze-Fan Chao, Gregory Y. H. Lip; Asia-Pacific Heart Rhythm Society Atrial Fibrillation Registry Investigators§.

Supplementary data

Supplementary Table 1. Multivariate analysis for factors associated with Vitamin K Antagonist use.

|  | HR | 95%CI | p-value |
| --- | --- | --- | --- |
| Age | 0.98 | 0.97-0.99 | <0.001 |
| Female sex | 0.99 | 0.81-1.21 | 0.944 |
| CHA_2_DS_2_-VASc | 1.22 | 1.13-1.31 | <0.001 |
| Paroxysmal AF | 0.43 | 0.35-0.52 | <0.001 |
| CKD | 2.03 | 1.50-2.76 | <0.001 |
| Cancer | 0.42 | 0.21-0.84 | 0.015 |
| Dementia | 1.50 | 0.77-1.73 | 0.231 |
| Anemia | 2.72 | 1.95-3.79 | <0.001 |
| Previuos bleeding | 0.90 | 0.64-1.27 | 0.548 |
| COPD | 1.11 | 0.66-1.88 | 0.685 |
| Liver disease | 1.15 | 0.77-1.73 | 0.482 |

HR: Hazard Ratio, CI: Confidence Interval, AF: Atrial Fibrillation, CKD: Chronic Kidney Disease; COPD: Chronic Obstructive Pulmonary Disease.

| Supplementary Table 2. Multivariate Cox-regression analysis for all-cause death. |
| --- |
| \|  \| HR \| 95%CI \| p-value \| \| --- \| --- \| --- \| --- \| \| Age \| 1.06 \| 1.04-1.09 \| <0.001 \| \| Female sex \| 0.67 \| 0.44-1.01 \| 0.054 \| \| CHA_2_DS_2_-VASc \| 1.46 \| 1.28-1.69 \| <0.001 \| \| Paroxysmal AF \| 0.53 \| 0.35-0.81 \| 0.003 \| \| OAC \| 0.47 \| 0.30-0.68 \| <0.001 \| \| CKD \| 1.81 \| 1.17-2.81 \| 0.008 \| \| Cancer \| 3.06 \| 1.71-5.47 \| <0.001 \| \| Dementia \| 1.65 \| 0.88-3.07 \| 0.118 \| \| Dyslipidemia \| 1.31 \| 0.89-1.92 \| 0.170 \| \| COPD \| 3.57 \| 2.04-6.26 \| <0.001 \| |

HR: Hazard Ratio, CI: Confidence Interval, AF: Atrial Fibrillation, CKD: Chronic Kidney Disease; COPD: Chronic Obstructive Pulmonary Disease.

| Supplementary Table 3. Multivariate Cox-regression analysis for cardiovascular death.   \|  \| HR \| 95%CI \| p-value \| \| --- \| --- \| --- \| --- \| \| Age \| 1.04 \| 0.99-1.10 \| 0.130 \| \| Female sex \| 0.80 \| 0.34-1.85 \| 0.594 \| \| CHA_2_DS_2_-VASc \| 1.54 \| 1.14-2.07 \| 0.005 \| \| Paroxysmal AF \| 0.31 \| 0.11-0.92 \| 0.034 \| \| OAC \| 1.00 \| 0.33-3.03 \| 1.000 \| \| CKD \| 1.49 \| 0.54-4.09 \| 0.440 \| \| Cancer \| 1.08 \| 0.15-8.03 \| 0.940 \| \| Dementia \| 4.31 \| 1.52-12.19 \| 0.006 \| \| Dyslipidemia \| 2.18 \| 0.90-5.29 \| 0.085 \| \| COPD \| 2.55 \| 0.58-11.21 \| 0.214 \|   HR: Hazard Ratio, CI: Confidence Interval, AF: Atrial Fibrillation, CKD: Chronic Kidney Disease; COPD: Chronic Obstructive Pulmonary Disease. |
| --- | --- | --- | --- | --- | --- | --- | --- | --- | --- | --- | --- | --- | --- | --- | --- | --- | --- | --- | --- | --- | --- | --- | --- | --- | --- | --- | --- | --- | --- | --- | --- | --- | --- | --- | --- | --- | --- | --- | --- | --- | --- | --- | --- | --- |

| Supplementary Table 4. Multivariate Cox-regression analysis for thromboembolism.   \|  \| HR \| 95%CI \| p-value \| \| --- \| --- \| --- \| --- \| \| Age \| 1.04 \| 1.00-1.09 \| 0.077 \| \| Female sex \| 1.75 \| 0.76-4.04 \| 0.190 \| \| CHA_2_DS_2_-VASc \| 1.04 \| 0.75-1.42 \| 0.837 \| \| Paroxysmal AF \| 0.76 \| 0.35-1.66 \| 0.486 \| \| OAC \| 0.42 \| 0.18-0.98 \| 0.044 \| \| CKD \| 0.98 \| 0.28-3.42 \| 0.972 \| \| Cancer \| 1.21 \| 0.16-9.09 \| 0.851 \| \| Dementia \| 0.88 \| 0.11-6.95 \| 0.905 \| \| Dyslipidemia \| 1.64 \| 0.76-3.56 \| 0.210 \| \| COPD \| 1.37 \| 0.18-10.56 \| 0.761 \|   HR: Hazard Ratio, CI: Confidence Interval, AF: Atrial Fibrillation, CKD: Chronic Kidney Disease; COPD: Chronic Obstructive Pulmonary Disease. |
| --- | --- | --- | --- | --- | --- | --- | --- | --- | --- | --- | --- | --- | --- | --- | --- | --- | --- | --- | --- | --- | --- | --- | --- | --- | --- | --- | --- | --- | --- | --- | --- | --- | --- | --- | --- | --- | --- | --- | --- | --- | --- | --- | --- | --- |

| Supplementary Table 5. Multivariate Cox-regression analysis for acute coronary syndrome.   \|  \| HR \| 95%CI \| p-value \| \| --- \| --- \| --- \| --- \| \| Age \| 1.01 \| 0.98-1.05 \| 0.543 \| \| Female sex \| 0.52 \| 0.25-1.08 \| 0.080 \| \| CHA_2_DS_2_-VASc \| 1.27 \| 0.99-1.63 \| 0.059 \| \| Paroxysmal AF \| 1.35 \| 0.72-2.52 \| 0.349 \| \| OAC \| 0.47 \| 0.23-0.95 \| 0.034 \| \| CKD \| 1.17 \| 0.44-3.09 \| 0.751 \| \| Cancer \| - \| - \| - \| \| Dementia \| 1.71 \| 0.39-7.44 \| 0.477 \| \| Dyslipidemia \| 1.99 \| 1.02-3.87 \| 0.044 \| \| COPD \| 0.72 \| 0.10-5.32 \| 0.745 \|   HR: Hazard Ratio, CI: Confidence Interval, AF: Atrial Fibrillation, CKD: Chronic Kidney Disease; COPD: Chronic Obstructive Pulmonary Disease. |
| --- | --- | --- | --- | --- | --- | --- | --- | --- | --- | --- | --- | --- | --- | --- | --- | --- | --- | --- | --- | --- | --- | --- | --- | --- | --- | --- | --- | --- | --- | --- | --- | --- | --- | --- | --- | --- | --- | --- | --- | --- | --- | --- | --- | --- |

| Supplementary Table 6. Multivariate Cox-regression analysis for heart failure.   \|  \| HR \| 95%CI \| p-value \| \| --- \| --- \| --- \| --- \| \| Age \| 1.01 \| 0.98-1.02 \| 0.966 \| \| Female sex \| 0.95 \| 0.61-1.49 \| 0.823 \| \| CHA_2_DS_2_-VASc \| 1.37 \| 1.17-1.60 \| <0.001 \| \| Paroxysmal AF \| 0.50 \| 0.31-0.79 \| 0.004 \| \| OAC \| 0.86 \| 0.50-1.49 \| 0.597 \| \| CKD \| 1.99 \| 1.18-3.36 \| 0.010 \| \| Cancer \| 1.39 \| 0.51-3.82 \| 0.519 \| \| Dementia \| 1.83 \| 0.78-4.29 \| 0.163 \| \| Dyslipidemia \| 1.36 \| 0.89-2.09 \| 0.154 \| \| COPD \| 3.12 \| 1.50-6.66 \| 0.003 \|   HR: Hazard Ratio, CI: Confidence Interval, AF: Atrial Fibrillation, CKD: Chronic Kidney Disease; COPD: Chronic Obstructive Pulmonary Disease. |
| --- | --- | --- | --- | --- | --- | --- | --- | --- | --- | --- | --- | --- | --- | --- | --- | --- | --- | --- | --- | --- | --- | --- | --- | --- | --- | --- | --- | --- | --- | --- | --- | --- | --- | --- | --- | --- | --- | --- | --- | --- | --- | --- | --- | --- |

| Supplementary Table 7. Multivariate Cox-regression analysis for major bleeding.   \|  \| HR \| 95%CI \| p-value \| \| --- \| --- \| --- \| --- \| \| Age \| 1.05 \| 1.01-1.08 \| 0.005 \| \| Female sex \| 1.02 \| 0.55-1.87 \| 0.962 \| \| HAS-BLED \| 1.39 \| 1.02-1.89 \| 0.038 \| \| Paroxysmal AF \| 0.84 \| 0.45-0.58 \| 0.590 \| \| OAC \| 2.05 \| 0.73-5.77 \| 0.176 \| \| CKD \| 0.45 \| 1.13-1.56 \| 0.208 \| \| Cancer \| 2.20 \| 0.67-7.17 \| 0.193 \| \| Dementia \| 1.29 \| 0.30-5.58 \| 0.730 \| \| Dyslipidemia \| 1.02 \| 0.56-1.86 \| 0.950 \| \| COPD \| 1.25 \| 0.30-5.29 \| 0.763 \|   HR: Hazard Ratio, CI: Confidence Interval, AF: Atrial Fibrillation, CKD: Chronic Kidney Disease; COPD: Chronic Obstructive Pulmonary Disease. |
| --- | --- | --- | --- | --- | --- | --- | --- | --- | --- | --- | --- | --- | --- | --- | --- | --- | --- | --- | --- | --- | --- | --- | --- | --- | --- | --- | --- | --- | --- | --- | --- | --- | --- | --- | --- | --- | --- | --- | --- | --- | --- | --- | --- | --- |

**Asia-Pacific Heart Rhythm Society Atrial Fibrillation Registry Investigators:**

Hong Kong: Chun-Wah Siu David (Queen Mary Hospital).

Japan: Wataru Shimizu, Kenji Yodogawa (Department of Cardiovascular Medicine Graduate School of Medicine Nippon Medical School); Hiroyuki Tsutsui, Yasushi Mukai (Department of Cardiovascular Medicine, Faculty of Medical Sciences, Kyushu University); Hirofumi Tomita, Daisuke Horiuchi (Department of Cardiology, Hirosaki University Graduate School of School of Medicine); Joji Hagii (Hirosaki Stroke and Rehabilitation Center); Kazutaka Aonuma (Division of Cardiology, University of Tsukuba Hospital); Yasuo Okumura (Division of Cardiology, Nihon University Itabashi Hospital); Masahiko Goya, Kenzo Hirao (Department of Cardiovascular Medicine, Tokyo Medical and Dental University); Masayoshi Ajioka (Division of Cardiology, Tosei General Hospital); Nobuhisa Hagiwara, Atsushi Suzuki (Department of Cardiology, Tokyo Women's Medical University); Teiichi Yamane (Department of Cardiovascular Medicine, Jikei University); Takanori Ikeda, Hitomi Yuzawa (Toho University (Faculty of Medicine)); Kazuhiro Satomi, Yoshinao Yazaki (Heart Rhythm Center, Tokyo Medical University); Keiichi Fukuda(Department of Cardiology, Keio University School of Medicine); Yoshinori Kobayashi, Norishige Morita (Division of Cardiology, Tokai University Hachioji-hospital); Toyoaki Murohara (Department of Cardiology, Nagoya University); Eiichi Watanabe, Masahide Harada (Department of Cardiology, Fujita Health University School of Medicine); Satoru Sakagami, Takahiro Saeki (National Hospital Organization Kanazawa Medical Center); Kengo Kusano, Koji Miyamoto (Department of Cardiovascular Medicine, National Cerebral and Cardiovascular Center); Shinsuke Miyazaki, Hiroshi Tada (Department of Cardiovascular Medicine , Faculty of Medical Sciences, University of Fukui); Koichi Inoue, Nobuaki Tanaka(Cardiovascular center, Sakurabashi Watanabe Hospital); Yukihiro Koretsune, Haruhiko Abe (National Hospital Organization Osaka National Hospital, (Osaka, Japan); Yasuki Kihara, Yukiko Nakano (Department of Cardiovascular Medicine, Hiroshima University Graduate School of Biomedical and Health Sciences); Akihiko Shimizu, Yasuhiro Yoshiga (Department of Medicine and Clinical Science、University Graduate School of Medicine); Tomohiro Sakamoto, Ken Okumura(Division of Cardiology, Saiseikai Kumamoto Hospital Cardiovascular Center); Naohiko Takahashi, Tetsuji Shinohara (Oita University Hospital); Kyoko Soejima (Department of Cardiovascular Medicine, Kyorin University School of Medicine); Masahiko Takagi(Kansai Medical University Medical Center); Mitsuharu Kawamura, Yumi Munetsugu (Division of Cardiology, Showa University School of Medicine)

Korea: Sung-Hwan Kim (Division of Cardiology, Department of Internal Medicine, Seoul St. Mary's Hospital, College of Medicine, The Catholic University of Korea, Seoul, South Korea); Jae-Min Shim (Division of Cardiology, Korea University College of Medicine and Korea University Medical Center, Seoul, Republic of Korea); Jae Sun Uhm (Division of Cardiology, Department of Internal Medicine, Yongin Severance Hospital, Yonsei University College of Medicine, Yongin, Korea); Sung Il Im (Division of Cardiology, Department of Internal Medicine, Kosin University Gospel Hospital, Kosin University College of Medicine, Busan, Korea); Hyoung-Seob Par (Division of Cardiology, Department of Internal Medicine, Keimyung University Dongsan Hospital, Daegu, South Korea); Jun Hyung Kim (Department of Cardiology, Chungnam National University, Daejeon, Republic of Korea); Young Keun On (Division of Cardiology, Department of Medicine, Heart Vascular and Stroke Institute, Samsung Medical Center, Sungkyunkwan University School of Medicine); Il-Young Oh (Division of Cardiology, Department of Internal Medicine, Seoul National University Bundang Hospital); Seung Yong Shin (Cardiovascular & Arrhythmia Centre, Chung-Ang University Hospital, Chung-Ang University, Seoul, Korea); Jum Suk Ko (Division of Cardiology, Department of Internal Medicine, Wonkwang University School of Medicine, Iksan, Korea); Jun Beom Park (Department of Cardiology, College of Medicine, Ewha Womans University, Seoul, Korea)

Singapore: Wee-Siong Teo (National Heart Centre Singapore); Kelvin Cheok-Keng Wong (Changi General Hospital); Toon-Wei Lim (National University Hospital); David Foo (Tan Tock Seng Hospital)

Taiwan: Shih-Ann Chen (Taichung Veterans General Hospital); Shih-Ann Chen, Tze-Fan Chao, Yenn-Jiang Lin, Fa-Po Chung, Yu-Feng Hu, Shil-Lin Chang, Ta-Chuan Tuan, Jo-Nan Liao (Taipei Veterans General Hospital); Cheng-Hung Li, Jin-Long Huang, Yu-Cheng Hsieh, Tsu-Juey Wu, Ying-Chieh Liao (Taichung Veterans General Hospital); Cheng-Hung Chiang, Hsiang-Chiang Hsiao, Tung-Chen Yeh (Kaohsiung Veterans General Hospital); Wei-Siang Lin, Wen-Yu Lin (Tri-Service General Hospital); Jen-Yuan Kuo, Chong-Lie Hong, Yih-Je Wu, Ying-Siang Li, Jui-Peng Tsai, Kuo-Tzu Sung, Sheng-Hsiung Chang (Mackay Memorial Hospital).
